# Towards a standardization of non-symbolic numerical experiments: GeNEsIS, a flexible and user-friendly tool to generate controlled stimuli

**DOI:** 10.3758/s13428-021-01580-y

**Published:** 2021-06-11

**Authors:** Mirko Zanon, Davide Potrich, Maria Bortot, Giorgio Vallortigara

**Affiliations:** Center for Mind/Brain Sciences, University of Trento, Piazza Manifattura,1, 38068 Rovereto, Italy

**Keywords:** Number sense, Numerical abilities, Number comparison, Quantity discrimination, Approximate number system (ANS), Continuous physical variables, Standardized method, Numerical stimuli generator, MATLAB GUI

## Abstract

Several studies have suggested that vertebrate and invertebrate species may possess a *number sense*, i.e. an ability to process in a non-symbolic and non-verbal way the numerousness of a set of items. However, this hypothesis has been challenged by the presence of other non-numerical continuous physical variables, which vary along with numerosity (i.e., any change in the number of visual physical elements in a set naturally involves a related change in visual features such as area, density, contour length and convex hull of the stimulus). It is therefore necessary to control and manipulate the continuous physical information when investigating the ability of humans and other animals to perceive numerousness. During decades of research, different methods have been implemented in order to address this issue, which has implications for experiment replicability and inter-species comparisons, since no general standardized procedure is currently being used. Here we present the ‘Generation of Numerical Elements Images Software’ (GeNEsIS) for the creation of non-symbolic numerical arrays in a standardized and user-friendly environment. The main aim of this tool is to provide researchers in the field of numerical cognition a manageable and precise instrument to produce visual numerical arrays controlled for all the continuous variables. Additionally, we implemented the ability to actively guide stimuli presentation during habituation/dishabituation and dual-choice comparison tasks used in human and comparative research.

**Supplementary Information:**

The online version contains supplementary material available at 10.3758/s13428-021-01580-y.

## Introduction

The ability to operate with numerical information is an essential skill to deal with our everyday lives. However, the symbolic numerical system and the formal arithmetic developed by humans are believed to be rooted in a more ancient and possibly innate numerical ability, the so-called *number sense* (Dehaene, 1997). This sense of numbers is a non-symbolic, language-independent and evolutionarily conserved ability that allows humans and other animals to represent numerical sets of physical items in the surrounding environment (Ferrigno & Cantlon, 2017; Vallortigara, 2014, 2017). Animals, both vertebrates and invertebrates, can take advantage of this ability in order to optimize foraging decisions (vertebrates: Garland et al., 2012; Gazzola et al., 2018; Hanus & Call, 2007; Stancher et al., 2015; invertebrates: Bar-Shai et al., 2011; Hemptinne et al., 1992; Nelson & Jackson, 2012), conflicts between groups, defensive strategies (vertebrates: McComb et al., 1994; Potrich et al., 2015; Wilson et al., 2002; invertebrates: Tanner, 2006), mating competition (vertebrates: Flay et al., 2009; invertebrates: Carazo et al., 2009) and parental care (Lyon, 2003).

It has been hypothesized that the *number sense* is actually instantiated in an approximate number system (ANS), a non-verbal mechanism that allows one to estimate and represent the numerosity of sets of physical elements, which obeys Weber’s Law (Gallistel, 1990), i.e. as the ratio between the numbers to be discriminated increases, response times increase and accuracy decreases (e.g., discriminating 5 vs. 15, with a 0.33 ratio, is easier than discriminating 10 vs. 20, with a 0.5 ratio; Gallistel & Gelman, 1992; for general reviews see e.g. Butterworth, 1999; Hyde, 2011; Nieder & Dehaene, 2009). Evidence for a dedicated neural network for processing numerical information involving parietal and prefrontal cortical regions has been reported in humans (Arsalidou & Taylor, 2011; Piazza & Eger, 2016) and non-human primates (Wang et al., 2015). Furthermore, neurons that show tuned selectivity to specific numerousness have been described in both humans (see Nieder, 2016 for review) and non-human species, such as primates (Viswanathan & Nieder, 2013) and corvids (Ditz & Nieder, 2015; Wagener et al., 2018). Recently, evidence for neurons sensitive to numerousness has been reported in the dorso-central pallium of zebrafish (Messina et al., 2020a, 2020b).

The evidence supporting non-symbolic numerical estimation in a variety of species is robust; however, specifying the precise nature of what is actually estimated is challenging, since it has been argued that estimation may be guided by non-numerical continuous physical variables, such as the amount of stimulus extension in space (Leibovich et al., 2017). The debate regarding the existence of a *number sense* is rooted in a methodological issue for which it is empirically impossible to separate numerical information from all other continuous properties at once, making it difficult to study the non-symbolic numerosity processed in isolation from continuous magnitudes. In a natural environment, a change in numerosity of a set of items usually involves a change in the overall items’ properties (such as volume, area and perimeter) that co-varies with numbers: five objects normally occupy a larger space than three objects. At the same time, if the elements are displaced at the same distance one from the others, the global occupied field (known also as convex hull) will be different, and inversely, by pairing the global field, the larger group will present a higher density.

As an alternative to the *number sense theory,* some authors have suggested that numbers are estimated and compared by combining the different sensory cues comprising the numerical value using a sensory-integration-system (Gebuis et al., 2016). According to another hypothesis, numerosities and magnitudes would be processed holistically, thus arguing that it would be more appropriate to refer to a *sense of magnitude* than to a *sense of number* (Leibovich et al., 2017). Evidence supporting a broader processing of numerosity and continuous magnitudes has been reported by several studies. Using an algorithm capable of generating dot arrays in which continuous variables might be congruent or incongruent with the number of elements in the set, Gebuis and Reynvoet (2011, 2012) showed that the participants’ judgement was influenced by the visual properties of the elements’ arrays. Continuous magnitude appears to influence performance even with numerosities in the subitizing range (Leibovich et al., 2015; Salti et al., 2017). Studies carried out in humans and different animal species have shown that numerical tasks might be influenced by non-numerical cues such as the overall surface area (Feigenson et al., 2002; Stevens et al., 2007; Tokita & Ishiguchi, 2010), the individual size (Beran et al., 2008; Henik et al., 2017), the spatial frequency (Felisatti et al., 2020), and the density of the elements array (Dakin et al., 2011; Gómez-Laplaza & Gerlai, 2013).

This debate between *sense of number* and *sense of magnitude* is leading researchers to pay particular attention to the control of continuous physical variables. The simultaneous balancing of all these variables at the same time, however, is not viable: for instance, when the convex hull of the stimuli increases, the density decreases and vice versa; similarly, when the overall area of two sets of elements with different numerousness is balanced, their overall contour length would differ (Gebuis et al., 2014; Leibovich & Henik, 2014; Mix et al., 2002). It is therefore important to set up control conditions in which the different continuous physical variables are randomized and contrasted with numerousness as such to rule out their possible use as non-numerical cues that may guide the discrimination.

The most common solution to the issue of continuous physical variables is to use sets of numerical stimuli controlled, in turn, for some (and different) visual features. The principle strategy is to make these continuous variables uninformative cues for discriminative judgement.

The use of computerized methods to create visual sets of stimuli allows reasonable advantages. In the last years, new algorithms have been developed for this purpose (see for example De Marco & Cutini, 2020; DeWind et al., 2015; Guillaume et al., 2020; Salti et al., 2017; see Table 1 for a comparison). These algorithms are usually based on strict mathematical constraints that tolerate a certain amount of error, usually minimal (e.g. up to a ± 0.001% pixel tolerance limit; De Marco & Cutini, 2020). Mathematical calculation also allows a standardized procedure to evaluate the continuous variables in the group of elements; thus, the experimenter can study their impact on the subjects’ performance, as well as being provided with a general method that makes it possible to study intra- and inter-species differences. This minimizes the impact of biased errors and experimental procedure differences (De Marco & Cutini, 2020; Guillaume et al., 2020).

**Table 1:**
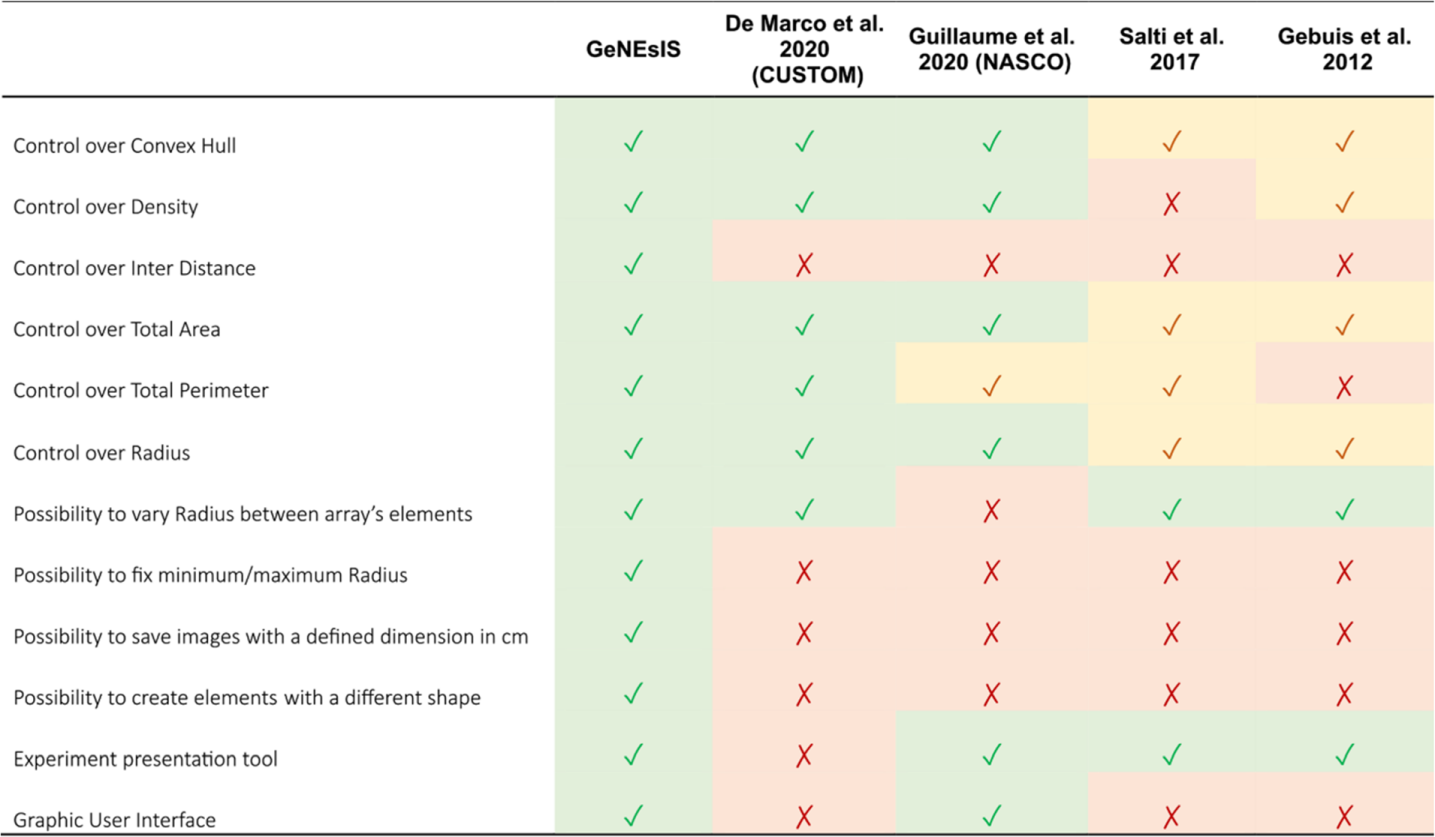
Comparison between GeNEsIS and other programs developed until now

The green check symbol indicates the ability of the program to perform the corresponding task, while the red cross indicates the absence of that tool. The yellow check indicates a limited possibility for the corresponding task: for instance, NASCO does not present the ability to select a custom total perimeter value, but since in this software the radius is fixed inside an array, the perimeter could be indirectly controlled; in Salti's software, instead, the controlled variables are not fully settable since the program only allows fixing their ratio across numerosities. With density, we refer here to a measure of mean occupancy, as slightly differently defined in the corresponding papers (De Marco & Cutini, 2020; Gebuis & Reynvoet, 2012; Guillaume et al., 2020; Salti et al., 2017), while with inter-distance we refer to the definition ‘most geometrically close to distance‘ that we gave (or similar definitions that can be found such as in De Marco and Cutini (2020), even if only presented in output and thus not directly controlled)

An example of these recent tools is given by Guillaume et al. (2020); despite taking into careful account the influence of several continuous variables, such as area, size, convex hull and each element’s spatial occupancy, the program does not allow proper control of the internal size of elements, producing arrays composed of identical items. This may cause important differences in terms of spatial frequency.

An alternative tool was developed by De Marco and Cutini (2020), allowing a flexible creation of dot arrays; however, this program lacks some important features, such as an active control of the items' inter-distance, the minimum and maximum distance that can divide neighbour elements, as well as a fixed minimum and/or maximum dimension of elements. Besides, this program does not present an interactive interface that allows a non-expert user to simply interact with the program.

The main goal of the algorithm we present here, GeNEsIS (*Generation of Numerical Elements Images Software,* GeNEsIS), would be to provide an alternative tool that integrates all the aforementioned features, helping in solving the issues related to the creation of numerical stimuli. The program will guide the user throughout the creation of sets of elements with a user-friendly interface, making it possible to freely utilize the output images for different purposes. Moreover, it provides a tool to perform the stimuli presentation on screen through two of the most common experimental methods: a habituation/dishabituation task and a simultaneous dual-choice discrimination task. We believe that our software is a step towards a standardization of stimulus presentation in the study of numerical cognition. It improves the way in which experiments can be designed and controlled with respect to previous programs, adding more flexibility and a wider handling spectrum on continuous physical variables on which the experimenter could act (see Table 1 for a schematic comparison between the main existing programs, and Fig. 5 for accuracy comparisons among the most recent ones).

## Materials and methods

We present a custom program (GeNEsIS) written in MATLAB (MATLAB R2019a, The MathWorks Inc., Natick, MA, USA), using Appdesigner. GeNEsIS is user-friendly, with a step-by-step tutorial; both the program code and the tutorial can be freely downloaded from https://github.com/MirkoZanon/GeNEsIS.

**Fig. 1:**
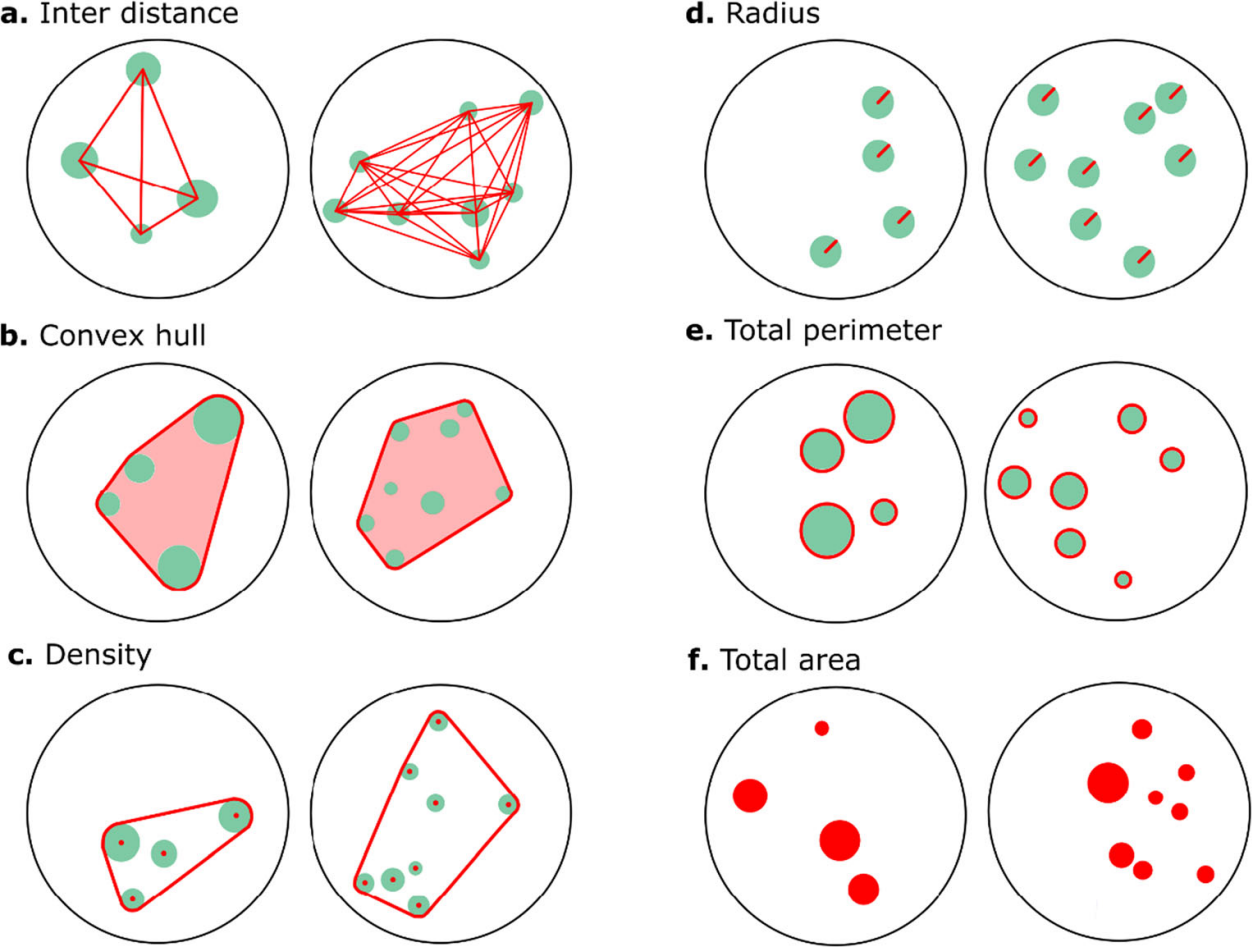
Example of stimuli with different numerosity (4 vs. 8 elements), controlled for the main continuous variables (sketched in red). **a** Inter-distance (ID): both groups are constructed with the same mean inter-distance between all elements. **b** Convex hull (CH): both groups are constructed with the same convex hull. **c** Density (D): both groups are constructed with the same density (n/CH). **d** Radius (R): both groups are constructed with all equal elements (fixed radius). **e** Total perimeter (TP): the sum of all the elements’ perimeters is the same for both groups. **f** Total area (TA): the sum of all the elements’ areas is the same for both groups. Combinations of one variable from the left group of controls (ID or CH or D) with one from the right (R or TP or TA) are also possible, allowing for maximum flexibility (see also Table 2 and Fig. 6)

Using GeNEsIS, the user can create stimuli with completely custom characteristics, precisely controlling all the relevant parameters and physical variables like convex hull, density, mean inter-distance, total surface area and total perimeter (see Fig. 1 for an example of each controllable variable; program accuracy in a stimuli generation sample is shown in the Supplementary Materials; see Fig. 1 Supplementary Materials). GeNEsIS is easy to use and allows great flexibility in stimuli creation. Moreover, the program implements a tool to perform automatized experiments using habituation/dishabituation and simultaneous discrimination procedures; the script can also be adapted and modified to fulfil different user necessities in presenting stimuli in many different ways. The presentation on screen exploits *Psychtoolbox-3* (the Psychophysics Toolbox extensions; Brainard, 1997; Kleiner et al., 2007; Pelli, 1997). Notably, the main tool to generate controlled stimuli is completely unrelated to any specific experiment or theory; thus it has a huge applicability spectrum and the creation of stimuli can be adapted to different theoretical frameworks.

**Table 2:** Summary of the main possible combinations between physical variables, as dictated by geometrical constraints

	**TA**	**TP**						
**ID**	TA+ID	TP+ID						
**D**	TA+D	TP+D						
**CH**	TA+CH	TP+CH		**R**_**min**_	**R**_**max**_	**R**_**min**_**+R**_**max**_	**R**_**equal**_	**R**_**free**_
			**TA+ID**	TA+ID+R_min_	TA+ID+R_max_	TA+ID+R_min_+R_max_	TA+ID+R_equal_	TA+ID+R_free_
			**TA+D**	TA+D+R_min_	TA+D+R_max_	TA+D+R_min_+R_max_	TA+D+R_equal_	TA+D+R_free_
			**TA+CH**	TA+CH+R_min_	TA+CH+R_max_	TA+CH+R_min_+R_max_	TA+CH+R_equal_	TA+CH+R_free_
			**TP+ID**	TP+ID+R_min_	TP+ID+R_max_	TP+ID+R_min_+R_max_	TP+ID+R_equal_	TP+ID+R_free_
			**TP+D**	TP+D+R_min_	TP+D+R_max_	TP+D+R_min_+R_max_	TP+D+R_equal_	TP+D+R_free_
			**TP+CH**	TP+CH+R_min_	TP+CH+R_max_	TP+CH+R_min_+R_max_	TP+CH+R_equal_	TP+CH+R_free_

We consider different combinations of inter-distance (ID), density (D), convex hull (CH), total area (TA), total perimeter (TP) and radius (R) (the last of which can be free to vary or equal inside an array; R_min_ and R_max_ refer to the ability to fix the minimum and maximum radius in the array). GeNEsIS can handle all these kinds of configurations, and even many other possible custom configurations that are less restrictive than these. The control of more variables at a time, different to the ones reported here, is not allowed given the geometrical limitations of these variables (i.e., their reciprocal dependency and their different covariation with numerosity)

The program works with MATLAB 2019 or more recent versions, and it has been thoroughly tested and proven to function in both Windows PC (e.g. Windows 10 Intel® Core™ i7-3770 - CPU: 3.4 GHz - RAM: 8 GB and Intel® Core™ i5-4570 - CPU: 3.2 GHz - RAM: 8 GB) and Mac (e.g. MacBook Pro Intel® Core™ i7 - CPU: 2.6 GHz - SSD: 512 GB - RAM: 16 GB and Intel® Core™ i5 - CPU: 2.7 GHz - RAM: 8 GB) environments.

### The controlled physical variables

GeNEsIS allows complete control over six main continuous variables (i.e. inter-distance, convex hull, density, radius, total perimeter and total area) and many other additional parameters (i.e., number of items in the array, shape and colour, arena type and dimension, accepted error and number of generations). Here we report a summary of all the parameters that the user can access and control, explaining how they are calculated.

- Inter-distance (ID): the average of the distances between all the possible pairs of elements (Fig. 1a). These are calculated as the Euclidean distances between the element centres. ID is not defined for one element.
- Convex hull (CH): the area of the smallest convex polygon containing all the elements (Fig. 1b); it is calculated through the *convhull()* MATLAB function using the points of all the elements’ perimeters. Thus, it can be seen as the surface area defined by the outermost stimuli perimeters. If only one element is presented, the CH value corresponds to its area.
- Density (D): the physical definition of elements’ density in a two-dimensional space, i.e. the number of elements (n) divided by the total occupied area (D = n/CH), (Fig. 1c). D is not defined for one element.
- Radius: the specific dimension of the elements. For the circular shapes it is the proper circle radius (Fig. 1d); for squares, diamonds and triangles it refers to the side dimension of the shapes.
- Total perimeter (TP): the sum of the perimeters (contours) of all elements (Fig. 1e).
- Total area (TA): the sum of the areas (surfaces) of all elements (Fig. 1f).

Additional controls of secondary variables can be applied, concerning the following points:

- Number of elements: number of elements in the visual array (the user is allowed to select 1 to *n* possible elements).
- Arena radius: the dimension of the field in which elements are created; it is possible to choose a circular or squared arena (in which case the term ‘radius’ refers to half the square side). The colour can be set using the RGB colour code.
- Shape and colour: the characteristics of elements that can be created, choosing between circles, squares, rotated squares (diamonds) and equilateral triangles. Completely custom colours can be set in RGB code.
- Accepted error: the tolerance of the program. Variables differing from the set value less than the ‘accepted error’ percent are considered to fulfil the requirements.
- Generations: the number of stimuli that are created at a time.

### GeNEsIS workflow

The program is structured in three steps: the computation of the stimuli with controlled physical variables (*GeNEsIS_create*), the saving of the final images with a custom layout (*GeNEsIS_save*) and the optional presentation of images on screen for experimental use (*GeNEsIS_display*).

#### First step: the computation of stimuli with controlled geometrical characteristics

**Fig. 2:**
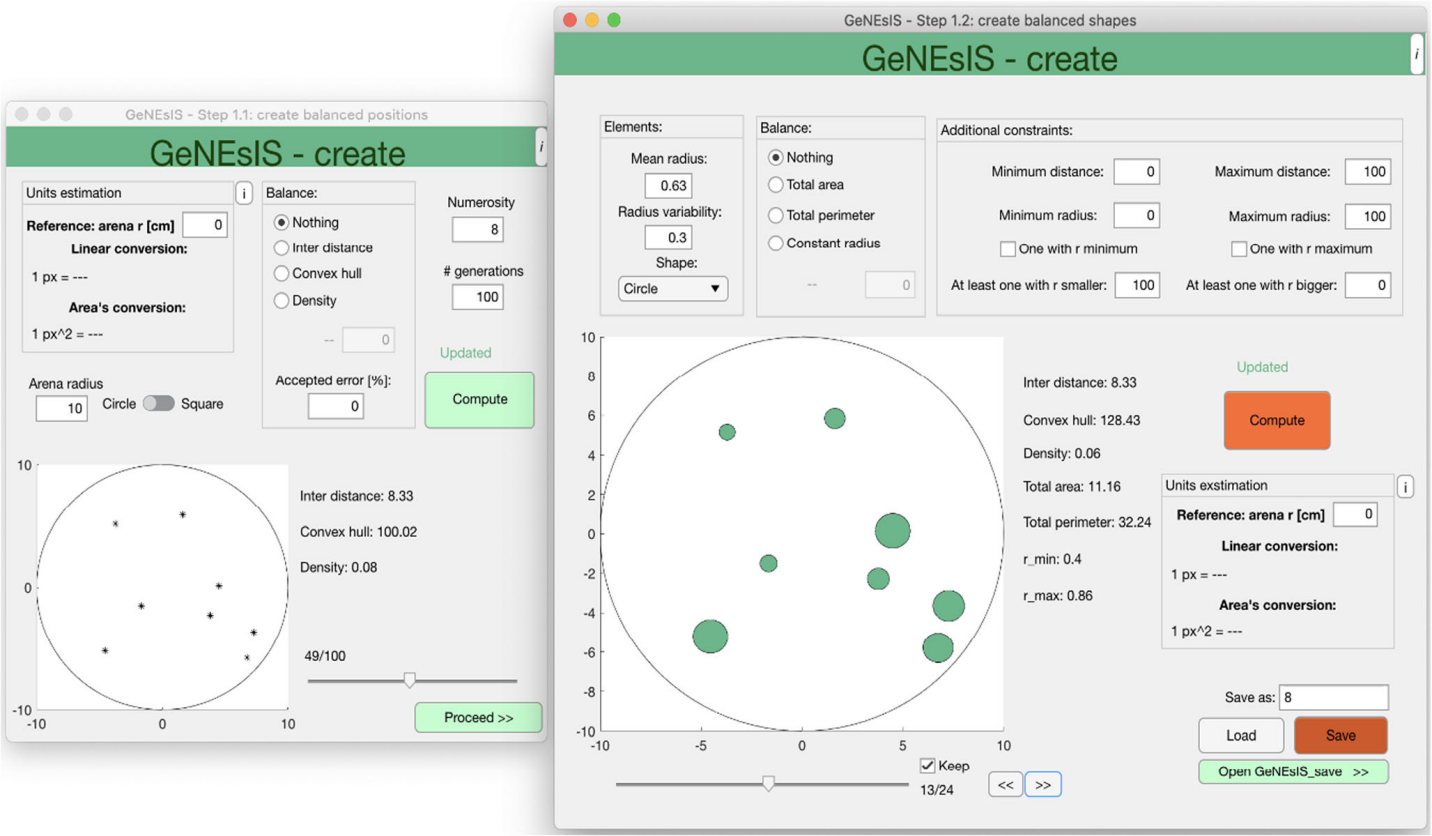
Graphic interface for the generation of GeNEsIS stimuli, (first step, GeNEsIS_create). In this step, the user can choose the continuous variables to control (ID, CH, D in the first passage -left-; R, TP, TA, shape in the second passage -right-). The program has an intuitive user-friendly interface to guide users through all the process of stimuli computation

This step is performed with *GeNEsIS_create*. Here the user will be guided through two passages (Fig. 2): the control of elements’ distribution and of their shape. The first step allows the creation of stimuli distribution in space (controlling points’ convex hull, density, inter-distance), while the second step allows the control of area, perimeter, type of shape, and finally to eventually adjust the shaped elements’ distribution to match the selected convex hull (see later for a detailed discussion).

In the first passage, after setting the arena dimension and shape (i.e., circular or squared arena), and the number of elements, the script will continuously generate configurations with these characteristics, keeping only the ones fulfilling the spatial distribution constraints. It will stop after collecting the selected number of generations. Later, it will further discard the ones that do not meet the chosen shape/geometrical constraints (see below). For this reason, it would be desirable to perform a big number of generations in order to obtain a final pool of a reasonable number of items. Since this could take a long time, an *‘accepted error’* option is settable in order to keep also configurations differing from the set constraints by an error smaller than the accepted one. Already, with an accepted error of 0.01%, there is a huge improvement in the computational time (for example, when applying this threshold, the computational speed more than doubles in most cases). As noted, all the variables are calculated here for point distributions.

In the second passage, the geometrical characteristics of the elements like shape, dimensions, areas and perimeters can be set. It is also possible to impose strong constraints on the radii. If the elements do not have a fixed radius, a mean radius and a variability can be chosen. The program will try to generate the elements with radii belonging to a Gaussian distribution with that mean and sigma (variability). Also, the minimum and maximum radius for the elements arrays are settable and can be maintained fixed across arrays (Fig. 6). Every time the user changes the parameters and re-computes the elements, a new random set fulfilling the user's constraints is generated on the same positions. A fundamental step to improve the program precision when controlling the CH is the refinement of the elements’ positions. If the user chooses a fixed CH, only the initial disposition of points (in the previous passage) will fulfil that value. However, when shaping the elements around these points, their extensions in space increase and so does the effective CH. For this reason, the ‘real’ CH (i.e., considering the shapes’ perimeters) is recalculated in this second passage, and the elements’ positions are moved by small steps towards their centre of mass until the desired CH is matched. In this way, GeNEsIS will give a more precise estimation of the CH (with a negligible error at the pixel level, related to the set tolerance), considering the physical extension of all the elements. If the user does not select a fixed CH, the program will simply calculate and update the effective CH value. Saving these data, a MATLAB GeNEsIS file is created, ready to be used to customize layout characteristics as reported in the second step.

#### Second step: saving the final images with custom layout

**Fig. 3:**
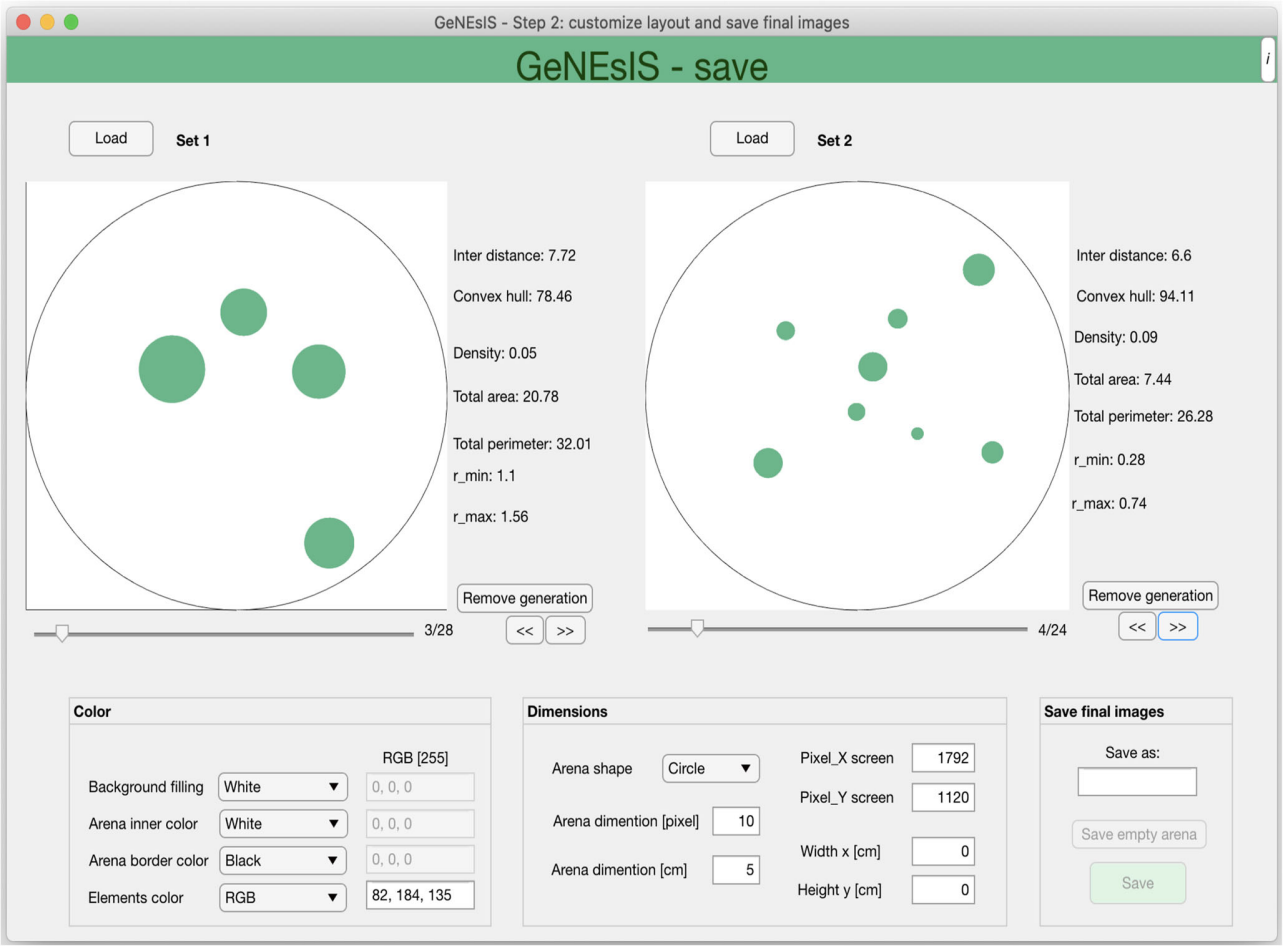
Interface for the graphic customisation of GeNEsIS stimuli, (second step, GeNEsIS_save). In this step, the user can choose all the possible colours and dimensions to save the final images. The stimuli can be saved as PNG images or MATLAB matrices ready to be used in a computerized presentation. The program has an intuitive user-friendly interface to guide through all the process of stimuli customization

This step is performed with *GeNEsIS_save*. After the creation of the stimuli pools, it is possible to save the created stimuli configurations as images ready to be presented during the experiment. Here, the effective dimensions, colours and other graphic characteristics can be chosen simultaneously for two different sets (Fig. 3). The tool allows for the greatest flexibility in colour customization, and moreover makes it possible to save different configurations of images as single or multiple stimuli in different orientations (horizontal or vertical). The output can be a PNG image file or a MATLAB matrix of images, ready to be used in an automated presentation with a computer. The elements’ dimensions in the output image can be precisely optimized, setting the output canvas dimension in pixels and the corresponding effective dimension in centimetres. In this way, fixing the desired effective dimension of the arena -taken as reference-, the user will obtain a figure with the exact final size. An Excel file is also saved in output as reference, with all the relevant characteristics of all the generated stimuli.

#### Third step: the presentation of stimuli on screen

**Fig. 4:**
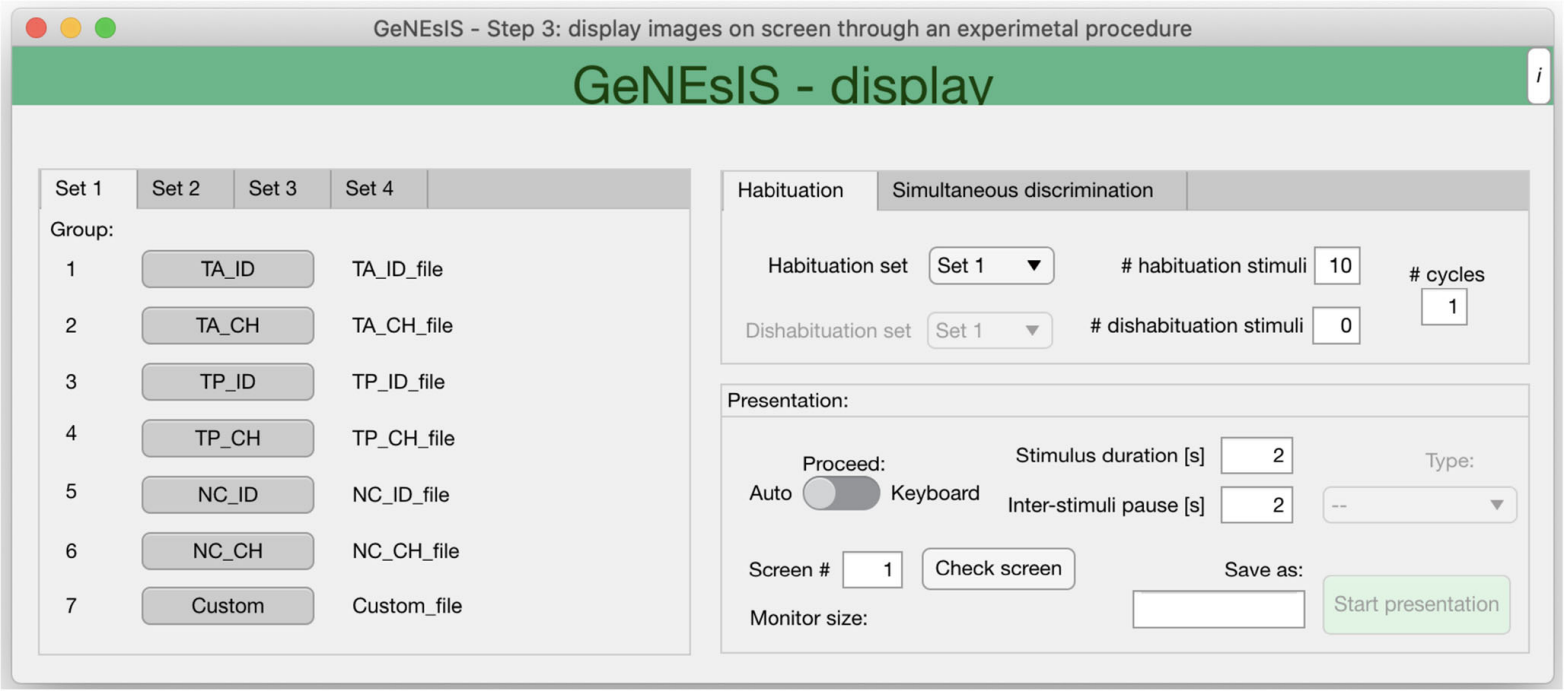
Interface for the performing of an experiment with GeNEsIS stimuli (third step, GeNEsIS_display). This is a tool allowing two classical experimental protocols: *habituation/dishabituation* and *dual choice* tasks. The program has an intuitive user-friendly interface to guide users through the whole experimental process and help in setting the presentation characteristics

 This step is performed with *GeNEsIS_display*. This supplementary tool, exploiting *Psychtoolbox*, allows the user to perform classical experiments on numerosity with the GeNEsIS stimuli (Fig. 4).

Two type of experimental paradigms can be implemented: the ‘habituation/dishabituation’ task and the ‘simultaneous dual-choice discrimination’ task, which are widely used in developmental and comparative cognition (Giurfa, 2019; Halberda & Feigenson, 2008; Messina et al., 2020b; Potrich et al., 2015; Xu & Spelke, 2000). The former implies the presentation of sets of stimuli whose physical properties all change from trial to trial, while maintaining the numerosity unchanged throughout the trials. During the dishabituation phase, novel stimuli with different characteristics (i.e., novel number of elements) are presented. The latter paradigm allows the user to display and visually compare two sets of stimuli that can differ in number. The stimuli to be compared can be balanced for one or more physical aspects and randomized among the session’s trials. The user can load different files created with GeNEsIS, up to four sets (numerosities) and choose the ones to present; the number of images per set is settable as well. In both experiments, the user can proceed to a subsequent trial, by pressing the keyboard or automatically, choosing the times of images presentation and pause. Again, all the sequences of stimuli presented during the experiments can be saved as an Excel file containing all relevant variables during the different presentation trials to help in the final evaluation of the experiment.

These are only two examples of experimental paradigms implemented using our program. The stimuli creation can be adapted for various needs and a large number of different experimental paradigms. Moreover, as reported above, the stimuli can also be saved as image files (PNG) and printed or used with other software (even different from GeNEsIS) to perform the final experiments.

## Results

### Comparison with existing tools

As previously reported, many available programs have different useful characteristics, but are not distilled into a suite of ideal tools for full control over the main continuous variables. With our software, we include all the main useful characteristics, adding additional features to refine the experimental design. We reported in Table 1 a sketched comparison of the main available programs developed so far (De Marco & Cutini, 2020; Gebuis & Reynvoet, 2012; Guillaume et al., 2020; Salti et al., 2017), focusing on the main tools a program could embed.

We also performed a comparison simulation with the two most recent (and most flexible, given the wider range of applications) programs (De Marco & Cutini, 2020; Guillaume et al., 2020). The outcome of the comparison is reported in Table 1.

The goal of the simulation was to create different arrays for three numerosities (5, 10, 20 elements), keeping constant the convex hull (controlled at 150000 pixels) and the total area (controlled at 20000 pixels) across quantities. In order to compare the simulations at the same level, and since NASCO (Guillaume et al., 2020) can only create dots with a constant radius inside an array, the array’s elements radius was kept constant in all simulations. For each numerosity, we created 100 images in order to collect the final output average and relative standard error. To test the fine precision of the different software, the error tolerance was set at 0.0001% for *GeNEsIS*, or the minimum possible reached by others.

**Fig. 5:**
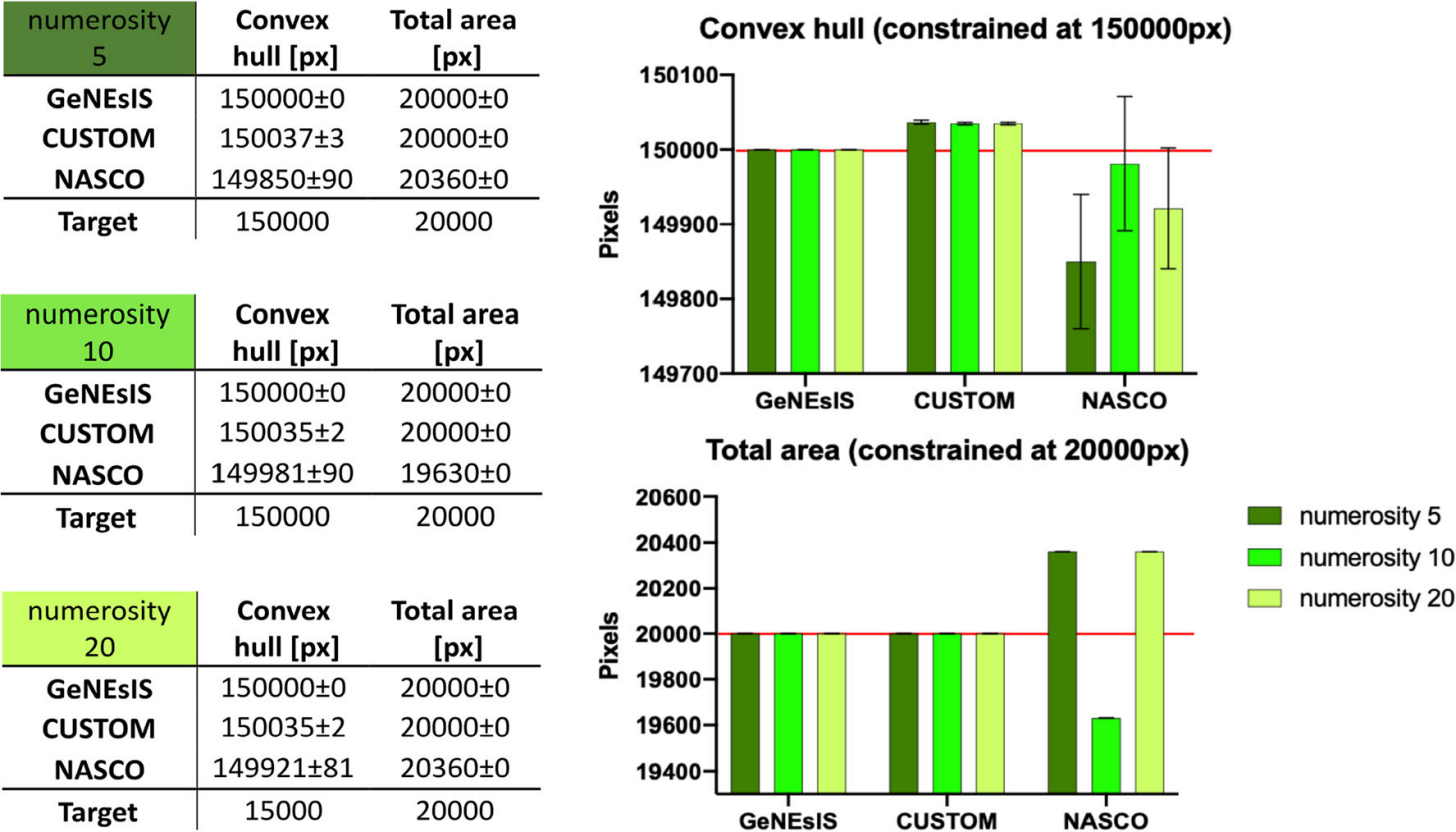
Comparison simulations between GeNEsIS and the most recent software (De Marco & Cutini, 2020; Guillaume et al., 2020). Three numerosities were generated (5, 10 and 20), with 100 images per numerosity. The aim was to fix the convex hull (at 150,000 px) and the total area (at 20,000 px) across numerosities. The tolerance was set at very low levels to test the fine precision of the software (0.0001% or the minimum possible). Means with relative standard errors for the controlled variables are reported in the tables and depicted in the bar graph as well for a direct comparison

The results are shown in Fig. 5, with the relative graphs representing the controlled features (convex hull and total area). In all the simulations, GeNEsIS showed high accuracy among the numerosities created, both when controlling the convex hull and when controlling the overall area.

It is interesting to note, across all these simulations, the very high accuracy of GeNEsIS (though the magnitude of errors is probably negligible in most cases; see Fig. 5).

We especially stress the usefulness of GeNEsIS for the wide range of tools it could provide compared with the other existing software (see Table 1) and the flexibility with which it can be used in many different contexts.

### Examples of stimuli creation for different theoretical frameworks

#### Physical variables controlled in combination

**Fig. 6:**
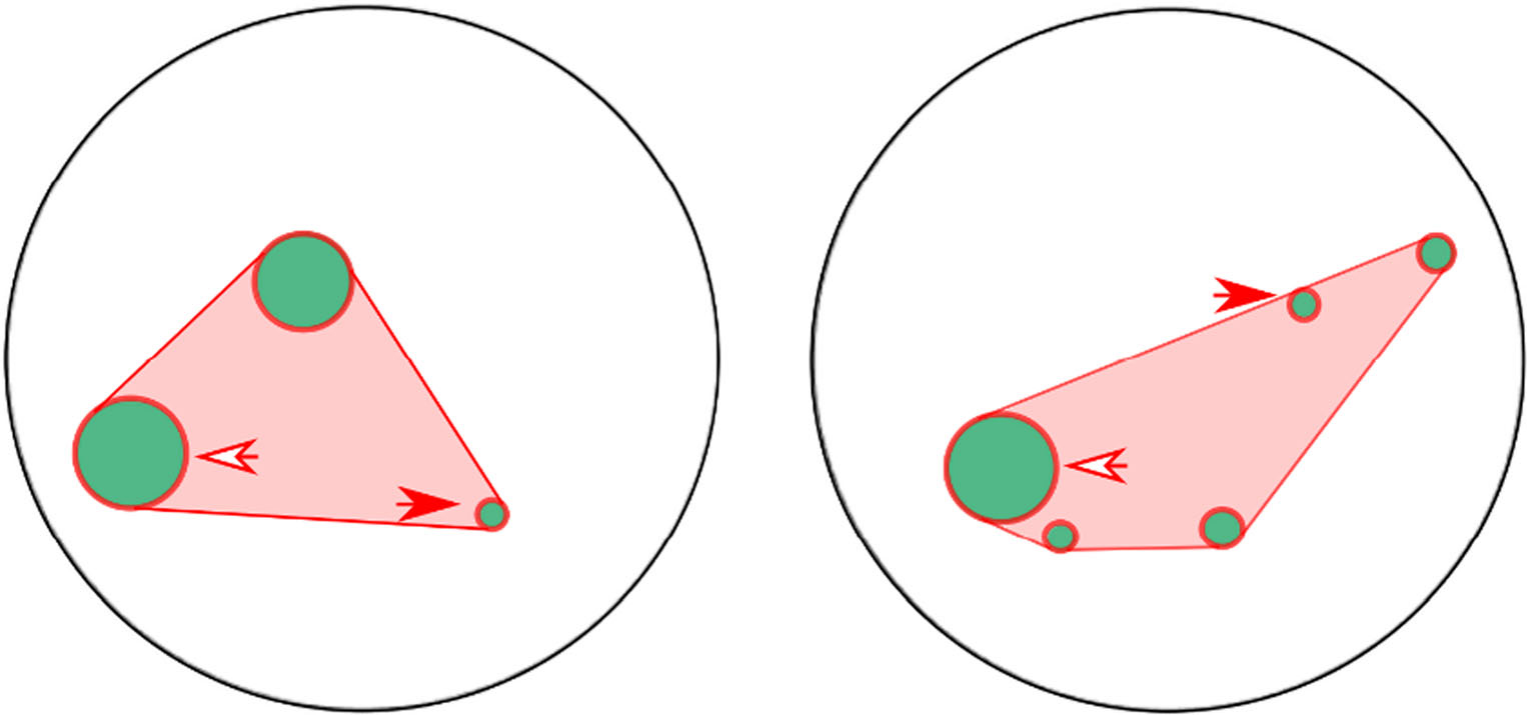
Example of stimuli with different numerosity (3 vs. 5 elements), controlled for a combination of different variables (sketched in red). In particular, both groups have the same convex hull and total perimeter. Moreover, a strict control over the radii is applied, imposing the same element size for the smallest and largest items (i.e. the radii of the smallest and largest elements are the same in both groups (see red and white arrows, respectively)

As previously reported, GeNEsIS controls different variables independently (see Fig. 1). Given the flexibility of the program, the user could also control custom combinations of these variables, with the only limitations given by the geometry and visual properties of the elements’ arrays (for example, it will never be possible to simultaneously fix total area and total perimeter given their geometrical characterization: as the first varies with the second power of the radius, the second varies linearly). A comprehensive overview is summarized in Table 2, in which the most significant variables are considered and coupled in accordance with their geometrical limitations. An example of multiple controls between numerosities 3 and 5 is also reported (Fig. 6).

Tests were additionally performed on the multiple control of different variables across numerosities, and all the main possible configurations were created. An exemplification of simulation for some of the most relevant combinations is reported in the Supplementary Material (Supplementary Fig. 1), where the accuracy of GeNEsIS in handling fixed variables and how the free variables vary across numerosities can be seen.

#### Creation of congruent and incongruent stimuli

**Fig. 7:**
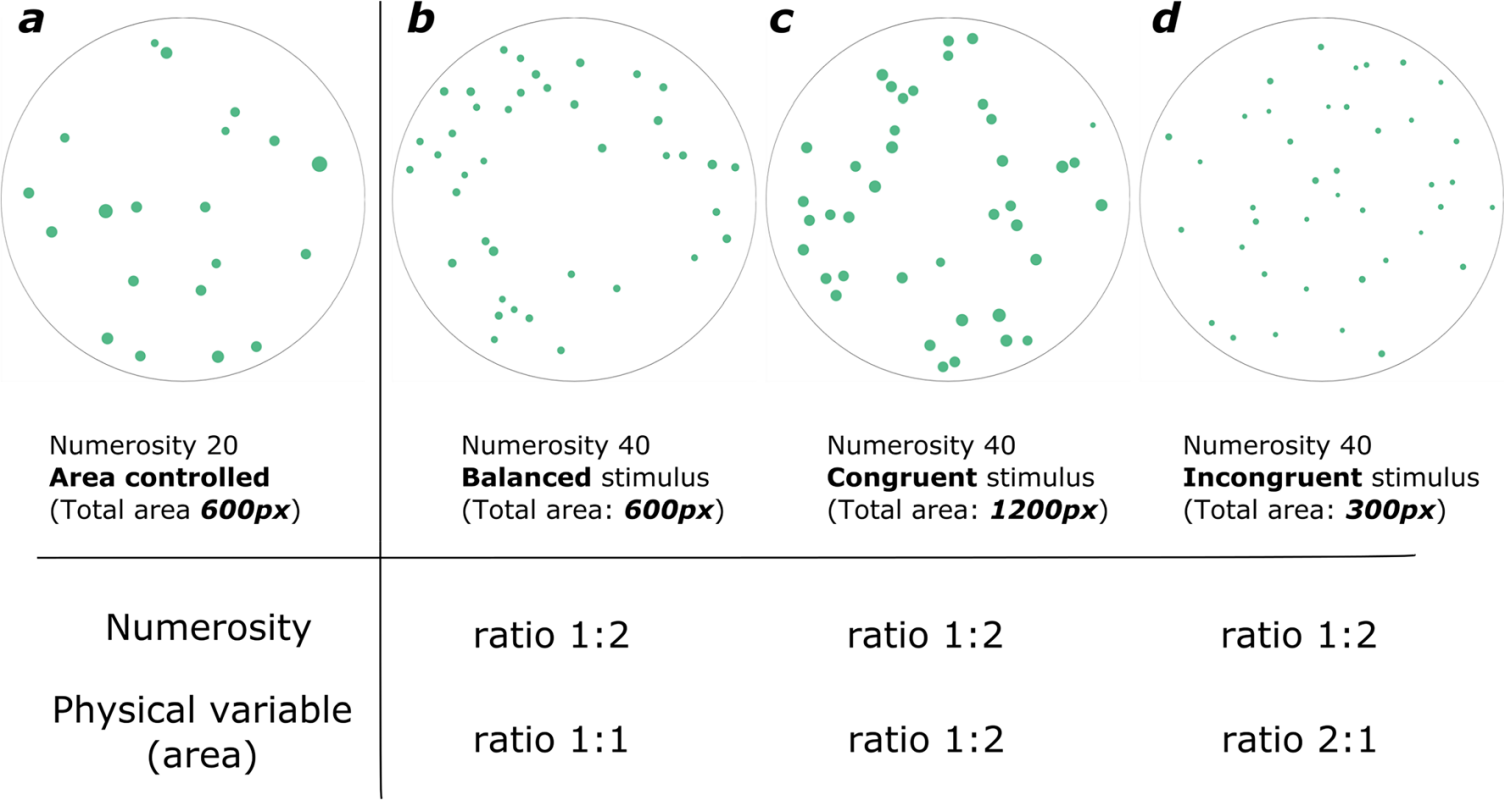
Example of comparison between 20 and 40 dots, controlled for their total area. **a** Numerosity 20 with fixed total area at 600 px. **b** Balanced stimulus of numerosity 40: total area is constant at 600 px. **c** Congruent stimulus of numerosity 40: total area (1200 px) doubles as numerosity doubles. **d** Incongruent stimulus of numerosity 40: total area (300 px) halves as numerosity doubles

Another possible use of GeNEsIS is given by the creation of sets of stimuli whose numerosity may vary congruently or incongruently with non-numerical physical variables. In order to report an example of this approach, we performed a simulation with 20 vs. 40 dots considering their total area as the continuous variable of interest. In Fig. 7a, the reference numerosity 20 is depicted with a fixed total area of 600 px; Fig. 7b shows a balanced stimulus with 40 dots, in which the numerosity doubles but the total area is still constant at 600 px (overall surface area is equal between the two numerosities). If instead one would create a congruent stimulus, this can be done by increasing the total area as the numerosity increases (Fig. 7c: the total area doubles with numerosity); on the contrary, it is possible to create incongruent stimuli by decreasing the overall area as the numerosity increases (Fig. 7d shows a stimulus incongruent for the total area).

This idea can be applied to every kind of variable and adapted to different theoretical backgrounds, showing the huge flexibility of GeNEsIS.

## Discussion and conclusions

Numerical abilities are important for many different species, since organisms use numerical information to perform adaptive choices (Haun et al., 2010; Nieder, 2020). However, researchers also identify alternative strategies to number encoding, based on perceptual cues that vary according to numerosity (Gebuis & Reynvoet, 2011, 2012); this raised the hypothesis of the existence of a more general *sense of magnitude* (Leibovich et al., 2017). Indeed, organisms could use the variation in continuous cues, such as area, perimeter or space occupied by elements, to discriminate arrays containing a different number of items.

For these reasons, a careful consideration of the variation in continuous physical variables when visual arrays are presented should be a mandatory aspect of investigation in numerical cognition. Despite previous studies having carefully applied several controls in the investigation of numerical abilities of different species, a common and standardized experimental method to guide such controls seems to be missing.

In the last few years, new programs have been developed to help researchers with numerosity element array creation (De Marco & Cutini, 2020; Guillaume et al., 2020; Salti et al., 2017). Despite their usefulness, each of them lacks some fundamental tool to allow full flexibility in creating controlled stimuli (see Table 1 for a comparison between programs). Here we propose GeNEsIS as a tool to create numerousness stimuli controlled in their physical continuous variables in a standardized and highly accurate (Fig. 5 and Supplementary Fig. 1) way. Our program sums up all the aforementioned utilities in a comprehensive tool, implementing the features that until now were missing (Table 1). In particular, with GeNEsIS the user can easily control convex hull, mean inter-distance, density, total area, total perimeter, elements’ number and size independently or in combination, with the only limitations imposed by the geometry (Table 2). Moreover, the layout is completely customizable, allowing the user to also create elements with different colours and shapes (circles, triangles, diamonds and squares). All the procedures are guided by an intuitive graphical user interface, permitting those even with less practical coding experience to easily create the stimuli. In addition, we implemented a tool to perform the presentation of these images on screen, during standardized experiments of habituation/dishabituation or dual choice tasks.

With this program we hope to fill the gap in the current state of the art in numerical cognition experiments, providing a tool that can improve the way in which experiments are conducted, reducing at a minimum the bias in numerousness array due to continuous physical variables, and standardizing the procedure of stimuli creation and presentation to facilitate experimental replicability and inter- and intra-species comparisons.

## Supplementary Information

ESM 1 (DOCX 473 kb)

## Data Availability

All the reported materials, data and the program code are freely available at https://github.com/MirkoZanon/GeNEsIS.

## References

[CR1] Arsalidou M, Taylor MJ (2011). Is 2+2=4? Meta-analyses of brain areas needed for numbers and calculations. NeuroImage.

[CR2] Bar-Shai N, Keasar T, Shmida A (2011). The use of numerical information by bees in foraging tasks. Behavioral Ecology.

[CR3] Beran MJ, Evans TA, Harris EH (2008). Perception of food amounts by chimpanzees based on the number, size, contour length and visibility of items. Animal Behaviour.

[CR4] Brainard, D. H. (1997). The Psychophsycis Toolbox. *Spatial Vision*, *10*(4), 433–436. 9176952

[CR5] Butterworth, B. (1999). *The mathematical brain*. Macmillan.

[CR6] Carazo P, Font E, Forteza-Behrendt E, Desfilis E (2009). Quantity discrimination in Tenebrio molitor: Evidence of numerosity discrimination in an invertebrate?. Animal Cognition.

[CR7] Dakin SC, Tibber MS, Greenwood JA, Kingdom FAA, Morgan MJ (2011). A common visual metric for approximate number and density. Proceedings of the National Academy of Sciences of the United States of America.

[CR8] De Marco D, Cutini S (2020). Introducing CUSTOM: A customized, ultraprecise, standardization-oriented, multipurpose algorithm for generating nonsymbolic number stimuli. Behavior Research Methods.

[CR9] Dehaene, S. (1997). *The number sense. How the mind creates mathematics*. Oxford University Press.

[CR10] DeWind NK, Adams GK, Platt ML, Brannon EM (2015). Modeling the approximate number system to quantify the contribution of visual stimulus features. Cognition.

[CR11] Ditz HM, Nieder A (2015). Neurons selective to the number of visual items in the corvid songbird endbrain. Proceedings of the National Academy of Sciences of the United States of America.

[CR12] Feigenson L, Carey S, Spelke E (2002). Infants’ discrimination of number vs. continuous extent. Cognitive Psychology.

[CR13] Felisatti, A., Laubrock, J., Shaki, S., & Fischer, M. (2020). A biological foundation for spatial–numerical associations: the brain’s asymmetric frequency tuning. *Annals of the New York Academy of Sciences*, *July*. 10.1111/nyas.14418 10.1111/nyas.14418 32645221

[CR14] Ferrigno, S., & Cantlon, J. F. (2017). Evolutionary Constraints on the Emergence of Human Mathematical Concepts. In: *Evolution of Nervous Systems, Second Edition* (Vol. 3, pp. 511–521). 10.1016/B978-0-12-804042-3.00099-3Evolution

[CR15] Flay CD, He XZ, Wang Q (2009). Influence of male density on the courtship and mating duration of male rice weevils, sitophilus oryzae. New Zealand Plant Protection.

[CR16] Gallistel, C. R. (1990). *Learning, development, and conceptual change. The organization of learning*. The MIT Press.

[CR17] Gallistel CR, Gelman R (1992). Preverbal and verbal counting and computation. Cognition.

[CR18] Garland A, Low J, Burns KC (2012). Large quantity discrimination by North Island robins (Petroica longipes). Animal Cognition.

[CR19] Gazzola A, Vallortigara G, Pellitteri-Rosa D (2018). Continuous and discrete quantity discrimination in tortoises. Biology Letters.

[CR20] Gebuis T, Cohen Kadosh R, Gevers W (2016). Sensory-integration system rather than approximate number system underlies numerosity processing: A critical review. Acta Psychologica.

[CR21] Gebuis T, Gevers W, Cohen Kadosh R (2014). Topographic representation of high-level cognition: Numerosity or sensory processing?. Trends in Cognitive Sciences.

[CR22] Gebuis T, Reynvoet B (2011). Generating nonsymbolic number stimuli. Behavior Research Methods.

[CR23] Gebuis T, Reynvoet B (2012). The interplay between nonsymbolic number and its continuous visual properties. Journal of Experimental Psychology: General.

[CR24] Giurfa M (2019). An Insect’s Sense of Number. Trends in Cognitive Sciences.

[CR25] Gómez-Laplaza LM, Gerlai R (2013). Quantification abilities in angelfish (Pterophyllum scalare): The influence of continuous variables. Animal Cognition.

[CR26] Guillaume, M., Schiltz, C., & Van Rinsveld, A. (2020). NASCO: A new method and program to generate dot arrays for non-symbolic number comparison tasks. *Journal of Numerical Cognition*, *6*(1), 129–147. 10.5964/jnc.v6i1.231

[CR27] Halberda J, Feigenson L (2008). Developmental Change in the Acuity of the “Number Sense”: The Approximate Number System in 3-, 4-, 5-, and 6-Year-Olds and Adults. Developmental Psychology.

[CR28] Hanus D, Call J (2007). Discrete Quantity Judgments in the Great Apes (Pan paniscus, Pan troglodytes, Gorilla gorilla, Pongo pygmaeus): The Effect of Presenting Whole Sets Versus Item-by-Item. Journal of Comparative Psychology.

[CR29] Haun DBM, Jordan FM, Vallortigara G, Clayton NS (2010). Origins of spatial, temporal and numerical cognition: Insights from comparative psychology. Trends in Cognitive Sciences.

[CR30] Hemptinne JL, Dixon AFG, Coffin J (1992). Attack strategy of ladybird beetles (Coccinellidae): factors shaping their numerical response. Oecologia.

[CR31] Henik A, Gliksman Y, Kallai A, Leibovich T (2017). Size Perception and the Foundation of Numerical Processing. Current Directions in Psychological Science.

[CR32] Hyde DC (2011). Two systems of non-symbolic numerical cognition. Frontiers in Human Neuroscience.

[CR33] Kleiner, M., Brainard, D., Pelli, D., Ingling, A., Murray, R., & Broussard, C. (2007). What's new in psychtoolbox-3. Perception, 36(14), 1-16.

[CR34] Leibovich T, Henik A (2014). Comparing performance in discrete and continuous comparison tasks. Quarterly Journal of Experimental Psychology.

[CR35] Leibovich T, Henik A, Salti M (2015). Numerosity processing is context driven even in the subitizing range: An fMRI study. Neuropsychologia.

[CR36] Leibovich, T., Katzin, N., Harel, M., & Henik, A. (2017). From “sense of number” to “sense of magnitude”: The role of continuous magnitudes in numerical cognition. *Behavioral and Brain Sciences*, *40*. 10.1017/S0140525X16000960 10.1017/S0140525X16000960 27530053

[CR37] Lyon BE (2003). Egg recognition and counting reduce costs of avian conspecific brood parasitism. Nature.

[CR38] McComb K, Packer C, Pusey A (1994). Roaring and numerical assessment in contests between groups of female lions, Panthera leo. Animal Behaviour.

[CR39] Messina, A., Potrich, D., Schiona, I., Sovrano, V. A., Fraser, S. E., Brennan, C. H., & Vallortigara, G. (2020a). *Neurons in the dorso-central division of zebrafish pallium respond to change in visual numerosity*. 1–34. 10.1101/2020.11.11.377804 10.1093/cercor/bhab218 PMC8754367 34322692

[CR40] Messina A, Potrich D, Schiona I, Sovrano VA, Fraser SE, Brennan CH, Vallortigara G (2020). Response to change in the number of visual stimuli in zebrafish:A behavioural and molecular study. Scientific Reports.

[CR41] Mix KS, Huttenlocher J, Levine SC (2002). Multiple cues for quantification in infancy: Is number one of them?. Psychological Bulletin.

[CR42] Nelson XJ, Jackson RR (2012). The role of numerical competence in a specialized predatory strategy of an araneophagic spider. Animal Cognition.

[CR43] Nieder A (2016). The neuronal code for number. Nature Reviews Neuroscience.

[CR44] Nieder, A. (2020). Absolute Numerosity Discrimination as a Case Study in Comparative Vertebrate Intelligence. *Frontiers in Psychology*, *11*. 10.3389/fpsyg.2020.01843 10.3389/fpsyg.2020.01843 PMC7426444 32849085

[CR45] Nieder A, Dehaene S (2009). Representation of number in the brain. Annual Review of Neuroscience.

[CR46] Pelli DG (1997). The VideoToolbox software for visual psychophysics: Transforming numbers into movies. Spatial Vision.

[CR47] Piazza M, Eger E (2016). Neural foundations and functional specificity of number representations. Neuropsychologia.

[CR48] Potrich D, Sovrano VA, Stancher G, Vallortigara G (2015). Quantity Discrimination by Zebrafish (Danio rerio). Journal of Comparative Psychology.

[CR49] Salti M, Katzin N, Katzin D, Leibovich T, Henik A (2017). One tamed at a time: A new approach for controlling continuous magnitudes in numerical comparison tasks. Behavior Research Methods.

[CR50] Stancher G, Rugani R, Regolin L, Vallortigara G (2015). Numerical discrimination by frogs (Bombina orientalis). Animal Cognition.

[CR51] Stevens JR, Wood JN, Hauser MD (2007). When quantity trumps number: Discrimination experiments in cotton-top tamarins (Saguinus oedipus) and common marmosets (Callithrix jacchus). Animal Cognition.

[CR52] Tanner CJ (2006). Numerical assessment affects aggression and competitive ability: A team-fighting strategy for the ant Formica xerophila. Proceedings of the Royal Society B: Biological Sciences.

[CR53] Tokita M, Ishiguchi A (2010). How might the discrepancy in the effects of perceptual variables on numerosity judgment be reconciled? MIDORI. Attention, Perception & Psychophysics.

[CR54] Vallortigara, G. (2015). Foundations of number and space representations in non-human species. In D.C. Geary, D. B. Bearch, & K. M. Koepke (Eds.), *Evolutionary origins and early development of number processing* (pp. 35–66). Elsevier. 10.1016/B978-0-12-420133-0.00002-8

[CR55] Vallortigara, G. (2017). An animal’s sense of number. In J. W. Adams, P. Barmby, & A. Mesoudi (Eds.), *The nature and development of mathematics. Cross disciplinary perspective on cognition, learning and culture* (pp. 43–65). Routledge.

[CR56] Viswanathan P, Nieder A (2013). Neuronal correlates of a visual “sense of number” in primate parietal and prefrontal cortices. Proceedings of the National Academy of Sciences of the United States of America.

[CR57] Wagener L, Loconsole M, Ditz HM, Nieder A (2018). Neurons in the endbrain of numerically naive crows spontaneously encode visual numerosity. Current Biology.

[CR58] Wang L, Uhrig L, Jarraya B, Dehaene S (2015). Representation of Numerical and Sequential Patterns in Macaque and Human Brains. Current Biology.

[CR59] Wilson ML, Britton NF, Franks NR (2002). Chimpanzees and the mathematics of battle. Proceedings of the Royal Society B: Biological Sciences.

[CR60] Xu F, Spelke ES (2000). Large number discrimination in 6-month-old infants. Cognition.

