# Supplementary material for: Towards a standardization of non-symbolic numerical experiments: GeNEsIS, a flexible and user-friendly tool to generate controlled stimuli
Source: Behav Res Methods. 2021 Jun 11;54(1):146–57. doi: 10.3758/s13428-021-01580-y (PMC8863760; doi:10.3758/s13428-021-01580-y)
Supplement: Supplementary file 1 — (DOCX 473 kb) [file 13428_2021_1580_MOESM1_ESM.docx]

# **Supplementary**


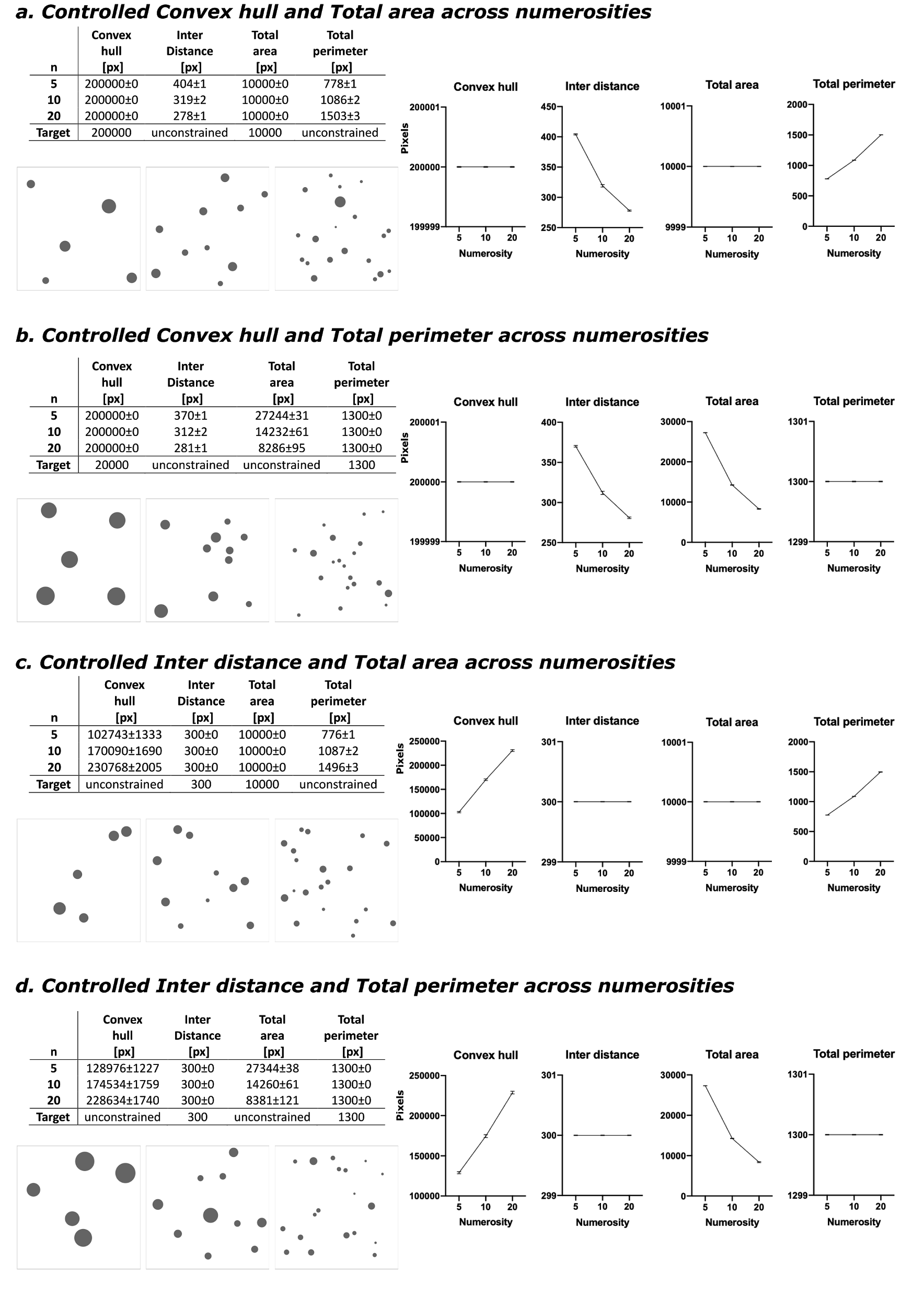


**Supplementary figure 1:** Example of the main combinations of variables controlled at a time, across different numerosities (5,10 and 20 elements). Four different combinations were tested (a. CH 200000px + TA 10000px; b. CH 200000px + TP 1300px; c. ID 300px + TA 10000px; d. ID 300px + TP 1300px). For each combination and each numerosity 100 images were created: results show the mean and standard error over these groups. In order to fine test the precision of GeNEsIS a small tolerance error was set (0.001%). The output mean results for the 4 main variables considered here are reported in the tables and in the relative graphs. One image per numerosity is also shown.
